# Gastroesophageal reflux disease over time in endoscopic versus surgical myotomy for treatment of achalasia: Systematic review and meta-analysis

**DOI:** 10.1055/a-2621-5421

**Published:** 2025-06-17

**Authors:** Angelo So Taa Kum, Beanie Conceição Medeiros Nunes, Eduardo Turiani Hourneaux Moura, Matheus Cavalcante Franco, Eduardo Guimarães Hourneaux de Moura

**Affiliations:** 1 Gastrointestinal Endoscopy Unit, Hospital das Clínicas da Faculdade de Medicina da Universidade de São Paulo, São Paulo, Brazil; 2 Endoscopy Unit, Hospital Sírio-Libanês, Brasília, Brazil

**Keywords:** Endoscopy Upper GI Tract, Reflux disease, Motility / achalasia, POEM, GI surgery

## Abstract

**Background and study aims:**

Achalasia, a motor disorder of the esophagus, is treated with peroral endoscopic myotomy (POEM) or Heller myotomy with fundoplication (HMF). Although POEM lacks an antireflux mechanism, potentially increasing postoperative gastroesophageal reflux disease (GERD), limiting the endoscopic approach, this difference in GERD tends to diminish over time. The objective of this study was to compare GERD over time, the need for regular gastric acid suppression therapy (GAST), effectiveness, and safety between POEM and HMF in treating achalasia.

**Methods:**

A systematic review and meta-analysis was conducted by searching mainly in MEDLINE, EMBASE, and ClinicalTrials.gov. Primary outcomes included early (< 12 months) and late (≥ 12 months) evaluations of postoperative GERD based on endoscopic criteria of the Lyon Consensus 2.0, and GAST. Secondary outcomes included clinical success (Eckardt score ≤ 3), procedure time, length of stay, and major adverse events (AEs) (Clavien-Dindo classification ≥ 2)

**Results:**

Thirty-two observational studies and two randomized trials were included, totaling 14,125 patients. GERD was higher in POEM in early evaluation (risk ratio [RR] = 3.03; *P* P < 0.01). GERD occurrence was similar between POEM and HMF in the late evaluation ( *P* = 0.30). Higher GAST was observed in POEM (RR = 1.35; *P* = 0.02). Secondly, clinical success was higher in POEM (RR = 1.06; *P* = 0.01), with shorter procedure time (median 39.53 minutes; *P* < 0.01) and shorter length of stay (mean different = -0.51 day; *P* = 0.03), with similar major AEs ( *P* = 0.81) compared with HMF.

**Conclusions:**

A higher rate of early GERD is observed in POEM compared with HMF, with the difference waning over time and reaching equivalence after 12 months, despite a higher rate of GAST in the endoscopic group. POEM also exhibited effectiveness and safety comparable to the surgical approach.

## Introduction

Achalasia is a pathology resulting from progressive degeneration of ganglion cells in the myenteric plexus in the esophageal wall associated with incomplete relaxation of the lower esophageal sphincter (LES). Its primary etiology is based on an idiopathic cause, with no defined factor, and secondary etiology is due to autoimmunity, resulting from the infiltration of T lymphocytes with destruction of ganglion cells, a hypothesis supported by a greater association with other autoimmune diseases; familial, such as Allgrove syndrome, an autosomal recessive disease characterized by adrenal insufficiency, alacrimia and esophageal achalasia; and infectious, secondary to herpes infection, measles and, mainly, Chagas disease, manifested by chronic infection by the parasite *Trypanosoma cruzi* . The main symptoms of achalasia are dysphagia, regurgitation, chest pain, weight loss, and atypical symptoms such as cough, odynophagia, and epigastric pain 1 2 3 .

The goal of treating achalasia is to reduce LES pressure, allowing food flow and improving symptoms, which may be surgical or medicinal. Current guidelines from the American Society for Gastrointestinal Endoscopy (ASGE) and the European Society for Gastrointestinal Endoscopy (ESGE) recommend treatment with pneumatic dilation, peroral endoscopic myotomy (POEM), and Heller myotomy with fundoplication (HMF) as the first option 4 5 . Both dilation and myotomy aim to reduce LES pressure by partial or total dissection of muscle fibers in the esophagus and cardia. However, among the three treatments, pneumatic dilation requires periodicity and recurrency of treatment to have the same effectiveness as POEM and HMF. Among the medications, use of calcium channel blockers and nitrates have been set aside due to side effects (headache, postural hypotension and edema) and short-term results associated with low effectiveness when compared with other methods 3 6 7 .

Ernst Heller first reported cardiomyotomy in 1914 8 and, once the technique was improved and established, Heller myotomy was associated with fundoplication, to become one of the main surgical therapies for treatment of achalasia 2 6 7 . Aiming for a less invasive approach, Haruhiro Inoue described his POEM technique as a new endoscopic treatment modality for achalasia in 2010, a procedure that consists of making an endoscopic submucosal tunnel from the mid-esophagus to the proximal stomach, followed by myotomy.

POEM combines the benefits of a theoretically less invasive endoscopic treatment with effectiveness and durability similar to HMF 3 6 7 . One of the main criticisms of the procedure is that it does not involve an antireflux method, which may lead to an increased rate of postoperative gastroesophageal reflux disease (GERD) compared with the surgical approach with fundoplication and compromising the endoscopic indication. However, a decreasing difference between the groups was observed over time 9 10 . GERD has typical symptoms such as heartburn, regurgitation, and retrosternal pain, and atypical symptoms such as eructation, cough, asthma, hoarseness, globus, nausea, abdominal pain, and dyspepsia. Of this range of symptoms, the typical symptoms of chest pain and regurgitation themselves resemble achalasia. With the aim of making a modern diagnosis, the Lyon Consensus 2.0 created objective criteria for diagnosis and exclusion of GERD to differentiate cardiac and pulmonary pathologies, esophageal motor disorders, and disorders of the gut-brain interaction (irritable bowel syndrome, functional dyspepsia and reflux hypersensitivity) 11 .

Given the evolving role of POEM as a minimally invasive alternative to HMF and concerns regarding long-term risk of GERD associated with POEM, a comparative evaluation of these two interventions is essential to guide clinical decision-making and optimize treatment strategies for achalasia. Applying the updated Lyon Consensus 2.0 11 , this systematic review and meta-analysis aimed to objectively evaluate and compare incidence of GERD over time, requirement for regular gastric acid suppression therapy (GAST), clinical effectiveness, and safety associated with POEM and HMF.

## Methods

### Protocol and registration

This systematic review and meta-analysis was conducted in accordance with the recommendations of the Cochrane Handbook for Systematic Reviews of Interventions 12 and the updated guidelines of the Preferred methodological tool Reporting Items for Systematic Reviews and Meta-Analyses (PRISMA), and included a completed PRISMA checklist ( **Supplementary Material 1** ) 13 . The study protocol was an update of a previous study 10 , which was registered in the international database PROSPERO ( https://www.crd.york.ac.uk/PROSPERO/ ) under registration number CRD42021259233.

### Source of information and literature search

A systematic review of data in the literature was performed with individualized searches of MEDLINE, EMBASE, Cochrane Library, and Clinicaltrials.gov from their inception to November 2024 by the first three authors. The following medical subject heading terms were used in each database: “(Heller Myotomy OR Heller OR Myotomy OR Cardiomyotomy OR Poem OR Peroral OR Per-oral OR Endoscopic OR Endoscopy) AND (Esophageal Achalasia OR Achalasia OR Achalasias OR Cardiospasm OR Megaesophagus)”.

### Study selection criteria and selected outcomes

Articles were included according to type: prospective or retrospective studies, observational or randomized controlled trials (RCTs), with availability of abstract and full text, regardless of date or language of publication; population: patients diagnosed with achalasia, regardless of subtype, etiology, age or previous treatment attempt; types of intervention: POEM versus HMF. Primary outcomes included assessment of GERD over time and outcomes that interfered, which included: early (< 12 months) and late (≥ 12 months) assessment of postoperative GERD using the updated and objective criteria from the Lyon Consensus 2.0 11 , and regular GAST to compare the percentage of patients in need of continuous medication. Because comparative studies between POEM and HMF that have pHmetry analysis used the outdated criteria for GERD by the DeMeester score 14 , following the Lyon Consensus 2.0 criteria 11 , this score does not meet the objective criteria and, consequently, was discarded for analysis. Therefore, the only valid objective parameter was based on the Lyon Consensus 2.0 endoscopic criteria by upper digestive endoscopy: Los Angeles Classification (LA) esophagitis grades B, C and D, Barrett's esophagus, and peptic esophageal stricture. Secondary outcomes evaluated the effectiveness, safety, and adverse events (AEs), which were clinical success, determined by the postoperative Eckardt symptom score ≤ 3 3 9 ; procedure time; length of hospital stay; and major AEs based on the Clavien-Dindo Classification grades 2 or more 15 . The following exclusion criteria were also applied: patients with secondary esophageal motility disorders; non-comparative studies between POEM and HMF; animal studies; and studies with incomplete data.

### Data extraction

All research results, abstracts, and full-text manuscripts were assessed for eligibility by three investigators who have experience in data extraction for retrospective and prospective studies, using predefined inclusion and exclusion criteria. If the same research group published more than one article, it was decided to include the most up-to-date data, and if there were different populations and complementary results, both studies were included. Data were entered into Microsoft Excel tables. Study data included the first author, year of publication, study design, period analyzed, sample size in each procedure, follow-up time, mean age, gender percentage, and outcomes.

### Risk of bias and quality of evidence

Internal validation and risk of bias in observational studies were performed using the Cochrane Risk Of Bias In Non-randomised Studies of Interventions (ROBINS-I) 16 . For randomized controlled trials (RCT), analysis was performed using the Cochrane risk-of-bias tool for RCTs (RoB-2) tool 17 . Quality of evidence was assessed using the Grading of Recommendations, Assessment, Development and Evaluations (GRADE) for each outcome using GRADEpro software - Guideline Development Tool 18 .

### Statistical analysis

Review Manager (RevMan) software version 5.4.1 from the Cochrane Collaboration was used to analyze outcomes. Effect sizes for continuous variables were analyzed using mean difference (MD) and standard deviation (SD) with a 95% confidence interval (CI). Risk ratio (RR) with a 95% confidence interval was used for categorical variables. RR and MD were statistically significant at a *P* ≤ 0.05. If a study provided medians and interquartile ranges, means and SD were extracted based on the McGrath method 19 . Heterogeneity between studies was assessed using the Tau² (τ ^2^ ) and the I ^2^ index introduced by the Higgins method 20 . Because Cochrane recommends exclusively using the random-effects model for meta-analyses, because it consistently accounts for underlying clinical and methodological variability among studies, regardless of statistical heterogeneity measures, the random-effect model was performed to analyze outcomes and the prediction interval (PI) was calculated 12 .

## Results

### Study selection and characteristics of included studies

The initial search identified 15,771 articles. After removing duplicate articles, screening by titles, abstracts, and applying the eligibility criteria, 34 studies were obtained for systematic review and meta-analysis, consisting of 32 observational studies 21 22 23 24 25 26 27 28 29 30 31 32 33 34 35 36 37 38 39 40 41 42 43 44 45 46 47 48 49 50 51 52 ( Table 1 ) and two RCTs 9 53 ( Table 2 ).

**Table:** **Table 1** Characteristics of included observational studies.

Study	Type	Period (year)	No. patients (POEM) No. patients (HMF)	Follow-up time (months)	Average age (years)	Male (%)
Akimoto S 2022 21	Retrospective Observational	1996 to 2019	14 11	11 72	58 51	50 36
Attaar M 2021 22	Prospective Observational	2010 to 2020	126 33	60 60	64 58	49 58
Bhayani NH 2014 23	Prospective Observational	2007 to 2012	37 64	≥ 6 ≥ 6	56 57	52 48
Caldaro T 2015 24	Prospective Observational	2009 to 2013	9 9	13 31	12 11	34 67
Chan SM 2016 25	Prospective Observational	2000 to 2014	33 23	6 60	48 38	37 48
Costantini A 2020 26	Prospective Observational	2014 to 2017	140 140	24 31	47 48	50 52
Docimo S Jr 2017 27	Retrospective Observational	2006 to 2015	44 122	NI	54 51	61 52
Fumagalli U 2016 28	Retrospective Observational	1996 to 2015	6 9	5 19	71 49	50 34
Greenleaf EK 2018 29	Retrospective Observational	2003 to 2016	20 21	11 65	60 58	60 48
Haider SA 2023 30	Retrospective Observational	2019 to 2020	15 15	12 12	54 53	47 47
Hanna AN 2018 31	Retrospective Observational	2011 to 2016	42 54	22 37	51 53	64 37
Hungness ES 2013 32	Prospective Observational	2004 to 2012	18 55	6	38 49	72 53
Kahaleh M 2020 33	Prospective Observational	2014 to 2019	69 64	12 12	47 46	42 47
Khashab MA 2017 34	Retrospective Observational	2009 to 2014	52 52	16 9	47 47	52 54
Khoraki J 2022 35	Retrospective Observational	2015 to 2018	1715 9555	NI	55 56	48 49
Kumagai K 2015 36	Prospective Observational	2012 to 2013	42 41	12 ≥ 6	46 45	64 54
Kumbhari V 2015 37	Retrospective Observational	2000 to 2013	49 26	9 22	58 52	59 50
Leeds SG 2017 38	Prospective Observational	2014 to 2017	12 11	12 10	52 53	33 54
Miller HJ 2017 39	Retrospective Observational	2011 to 2015	98 27	NI	NI	NI
Pascale S 2017 40	Retrospective Observational	2012 to 2015	32 42	24 27	56 48	37 55
Peng L 2017 41	Retrospective Observational	2009 to 2012	13 18	46 54	38 45	62 44
Podboy AJ 2021 42	Retrospective Observational	2010 to 2015	55 43	48 64	59 58	40 23
Ramirez M 2018 43	Prospective Observational	2010 to 2016	50 55	10 20	50 45	30 36
Schneider AM 2016 44	Retrospective Observational	2004 to 2016	25 25	8 36	50 54	52 48
Shally L 2023 45	Retrospective Observational	2014 to 2021	33 25	28 34	58 59	NI
Shea GE 2020 46	Retrospective Observational	2009 to 2018	44 97	18 45	52 52	60 60
Teitelbaum EN 2015 47	Prospective Observational	2013	36 20	11 12	50 53	69 45
Ujiki MB 2013 48	Prospective Observational	2009 to 2013	18 21	4 5	64 60	72 57
Ward MA 2017 49	Prospective Observational	2011 to 2015	41 24	≥ 12 ≥ 12	63 62	61 58
Ward MA 2021 50	Prospective Observational	2015 to 2019	54 46	10 10	57 54	35 28
Wirsching A 2019 51	Prospective Observational	2014 to 2017	23 28	6 6	58 57	48 43
Wong WJ 2023 52	Prospective Observational	2010 to 2021	63 60	18 18	42 48	46 52
HMF, Heller myotomy with fundoplication; NI, not informed; POEM, peroral endoscopic myotomy.						

**Table:** **Table 2** Characteristics of included randomized clinical trials.

Study	Type	Period (year)	No. patients (POEM) No. patients (HMF)	Follow-up time (months)	Average age (years)	Male (%)
de Moura ETH 2022 53	Randomized clinical trial	2017 to 2018	20 20	≥ 12 ≥ 12	45 44	40 30
Werner YB 2019 9	Randomized clinical trial	2012 to 2015	112 109	≥ 24 ≥ 24	49 49	61 55
HMF: Heller myotomy with fundoplication; POEM, peroral endoscopic myotomy.						

**Fig. 1:**
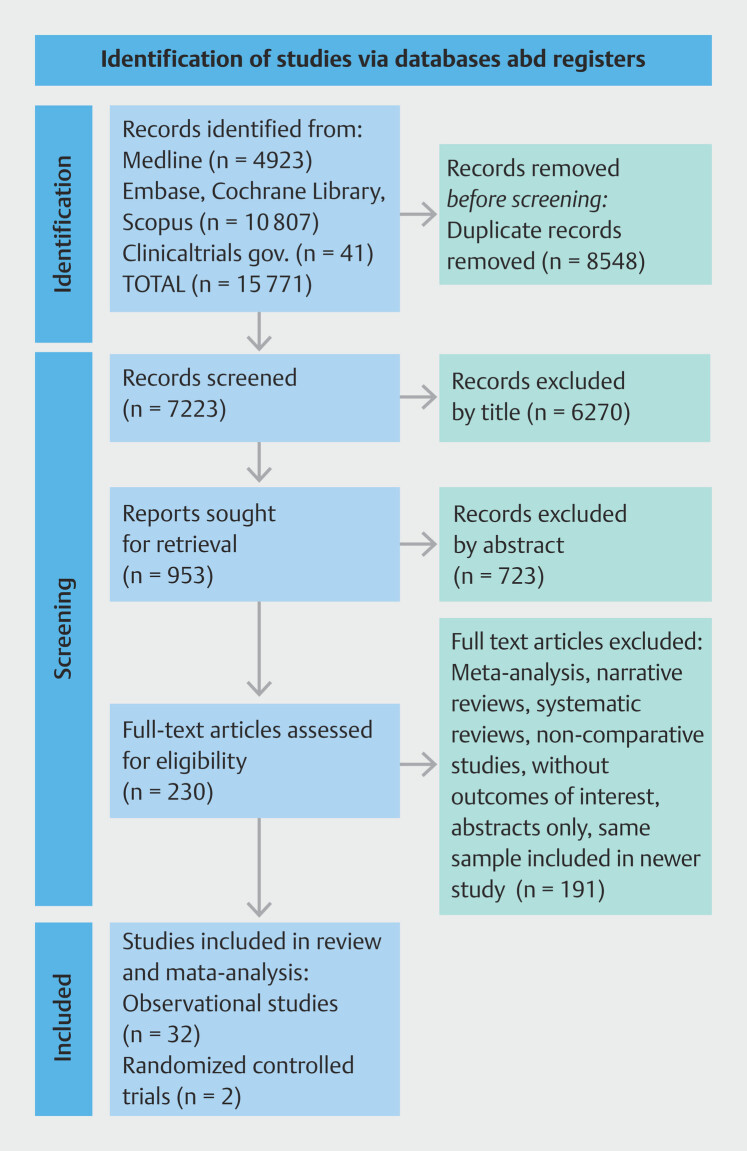
PRISMA diagram flowchart 13 .

The final PRISMA 13 diagram flowchart ( Fig. 1 ) resulted in 34 studies for systematic review and meta-analysis, evaluating 3,160 patients undergoing POEM and 10,965 undergoing HMF, totaling 14,125 patients.

### Risk of bias and quality of evidence

**Fig. 2:**
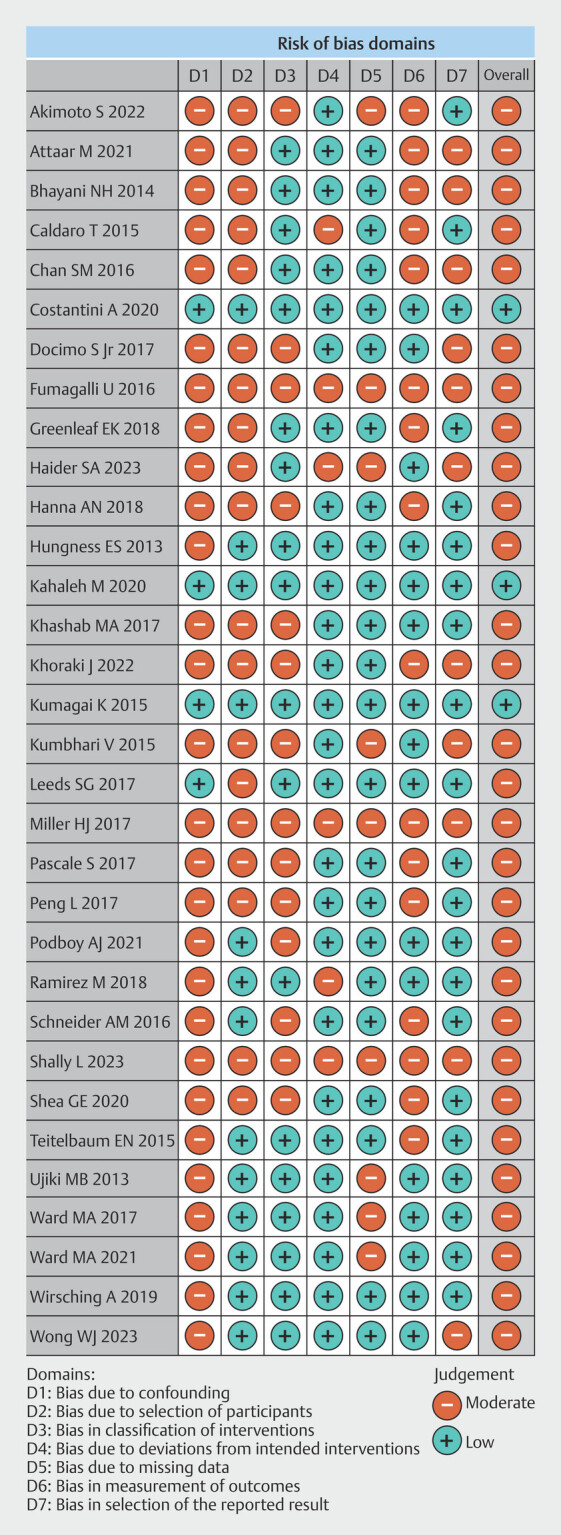
Risk of bias of observational studies by ROBINS-I.

**Fig. 3:**
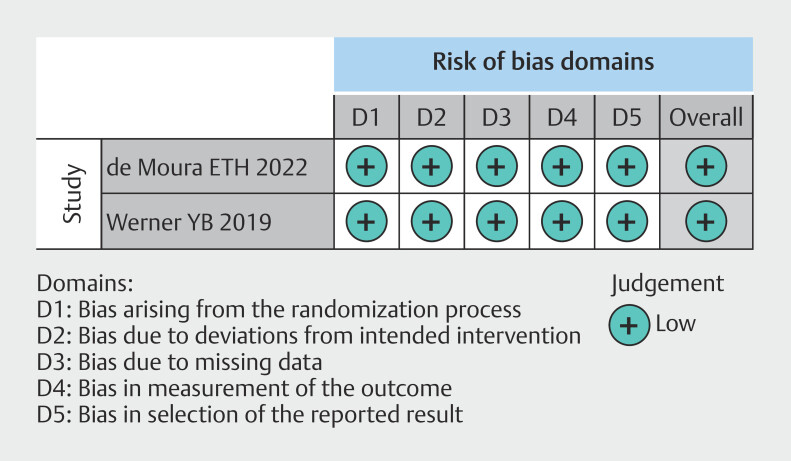
Risk of bias in randomized studies by RoB-2.

**Fig. 4:**
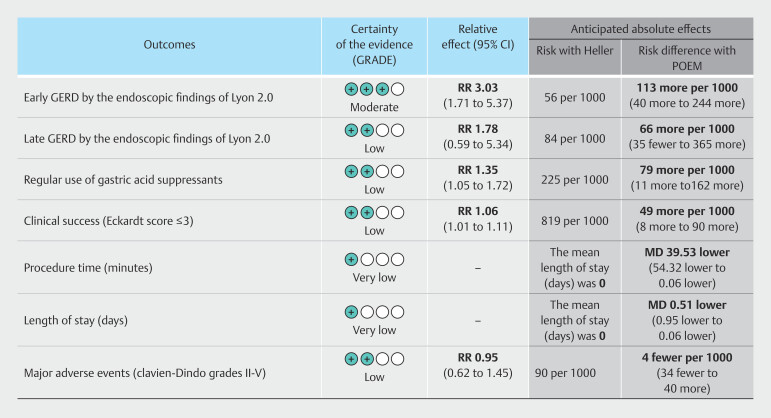
Quality of evidence assessed by GRADE.

Risk of bias among observational studies was moderate, except for Costantini et al. 26 , Kahaleh et al. 33 , and Kumagai et al. 36 , whose general bias risk were low ( Fig. 2 ). RCT data showed a low risk of bias ( Fig. 3 ). The quality of evidence assessed by GRADE is shown in Fig. 4 and will be discussed individually in the meta-analysis.

### Meta-analysis

#### Primary results

**Early GERD based on Lyon Consensus 2.0 endoscopic findings**

**Fig. 5:**
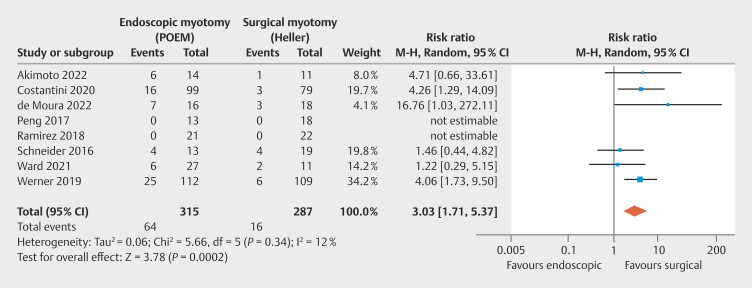
Forest plot of early GERD by Lyon Consensus 2.0 (< 12 months).

In early endoscopic findings, less than 12 months, adequate data were found in eight studies, consisting of six observational studies 21 26 41 43 44 50 and two RCTs 9 53 , totaling 602 patients. No significant heterogeneity was observed among the studies. Early GERD was higher in POEM compared with HMF, with statistical significance, favoring the surgical approach ( Fig. 5 : RR = 3.03; 95% CI 1.71–5.37; τ ^2^ = 0.06; I ^2^ =12%; PI = 1.342 to 6.840; *P* < 0.01), supported by a moderate quality of evidence by GRADE ( Fig. 4 ).

**Late GERD based on Lyon Consensus 2.0 endoscopic findings**

**Fig. 6:**
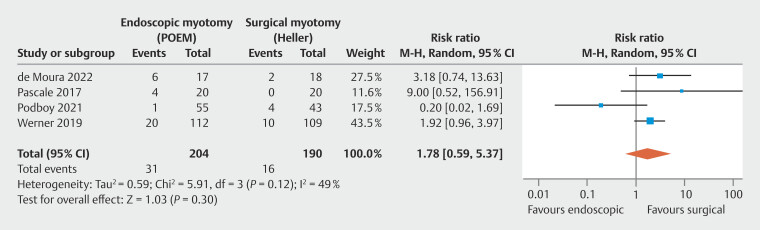
Forest plot of late GERD by Lyon Consensus 2.0 (≥ 12 months).

In late endoscopic findings, ≥ 12 months, adequate data were found in four studies, consisting of two observational studies 40 42 and two RCTs 9 53 , totaling 394 patients. No significant heterogeneity was observed among the studies. There was no difference in GERD between the groups ( Fig. 6 : RR = 1.78; 95% CI 0.59–5.34; τ ^2^ = 0.59; I ^2^ = 49%; PI = 0.122 to 25.992; *P* = 0.30), supported by a low quality of evidence by GRADE ( Fig. 4 ).

**Regular gastric acid suppression therapy**

**Fig. 7:**
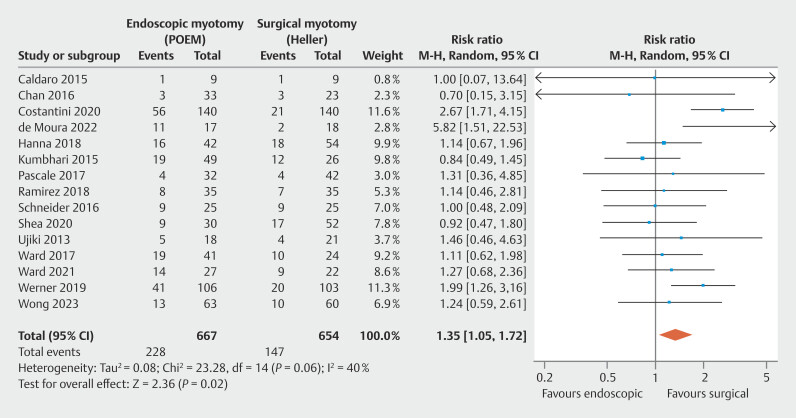
Forest plot of regular GAST.

Regarding continuous GAST, adequate data were found in 15 studies, consisting of 13 observational studies 24 25 26 31 37 40 43 44 46 48 49 50 52 and two RCTs 9 53 , totaling 1,321 patients. No significant heterogeneity was observed among the studies. There was a higher GAST needed in POEM compared with HMF, favoring the surgical approach, with statistical significance ( Fig. 7 : RR = 1.35; 95% CI 1.05–1.72; τ ^2^ = 0.08; I ^2^ = 40%; PI = 0.701–2.599; *P* = 0.02), supported by a low quality of evidence by GRADE ( Fig. 4 ).

#### Secondary results

**Clinical Success**

**Fig. 8:**
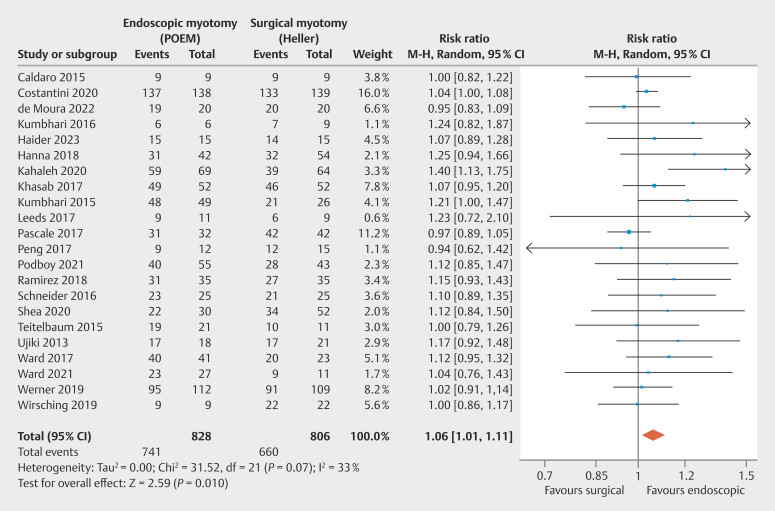
Forest plot of clinical success (Eckardt Score ≤ 3).

Adequate data were found in 22 studies evaluating the clinical success rate, consisting of 20 observational studies 24 26 28 30 31 33 34 37 38 40 41 42 43 44 46 47 48 49 50 51 and 2 RCTs 9 53 , totaling 1,634 patients. No significant heterogeneity was observed among the studies. Clinical success was higher in POEM compared with HMF, with statistical significance ( Fig. 8 : RR = 1.06; 95% CI 1.01–1.11; τ ^2^ = 0.00; I ^2^ = 33%; PI = 1.011–1.111; *P* = 0.01), supported by a low quality of evidence by GRADE ( Fig. 4 ).

**Procedure time**

**Fig. 9:**
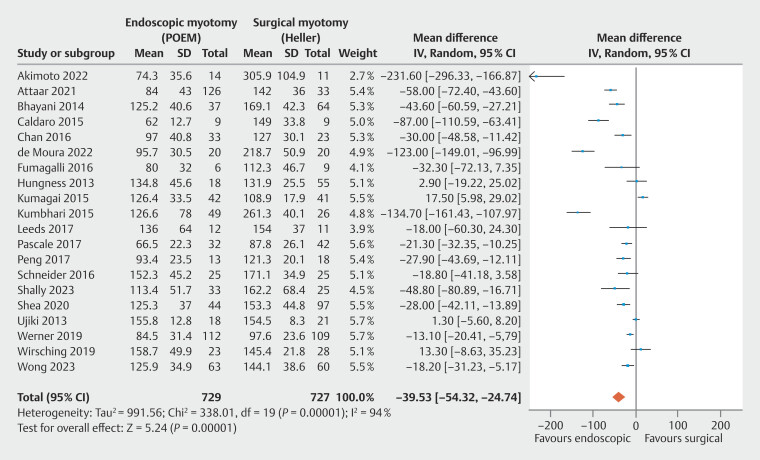
Forest plot of procedure time (minutes).

Adequate data were found in 20 studies evaluating procedure time, consisting of 18 observational studies 21 22 23 24 25 28 32 36 37 38 40 41 44 45 46 48 51 52 and two RCTs 9 53 , with a total of 1,456 patients. High heterogeneity was observed among the studies. POEM had shorter procedure time than HMF, with statistical significance ( Fig. 9 : MD = -39.53 minutes; 95% CI -54.32 to -24.74 minutes; τ ^2^ = 991.56; I ^2^ = 94%; PI = -107.076 to 28.016; *P* < 0.01), supported by a very low quality of evidence by GRADE ( Fig. 4 ).

**Length of hospital stay**

**Fig. 10:**
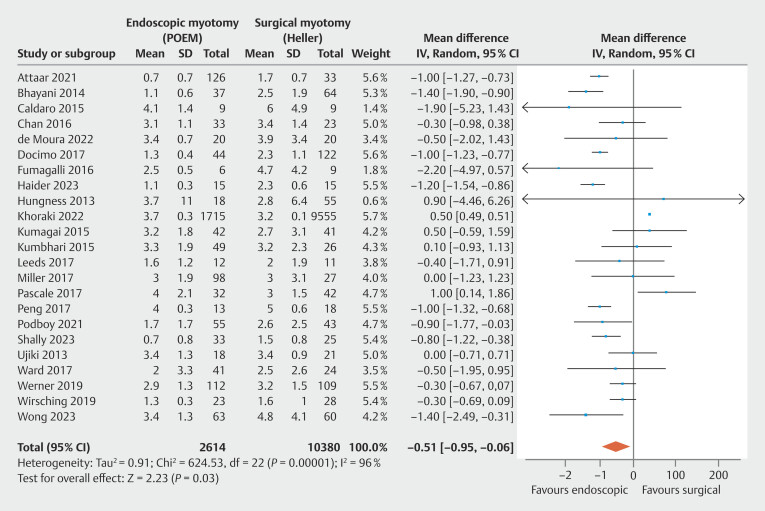
Forest plot of length of stay (days).

Adequate data were available from 23 studies, consisting of 21 observational studies 22 23 24 25 27 28 30 32 35 36 37 38 39 40 41 42 45 48 49 51 52 and two RCTs 9 53 , totaling 12,994 patients. High heterogeneity was observed among the studies. POEM had a shorter length of stay compared with HMF, with statistical significance ( Fig. 10 : MD = -0.51 day; 95% CI -0.95 to -0.06; τ ^2^ = 0.91; I ^2^ = 96%; PI = -2.538 to 1.518; *P* = 0.03), supported by a very low quality of evidence by GRADE ( Fig. 4 ).

**Major adverse events**

**Fig. 11:**
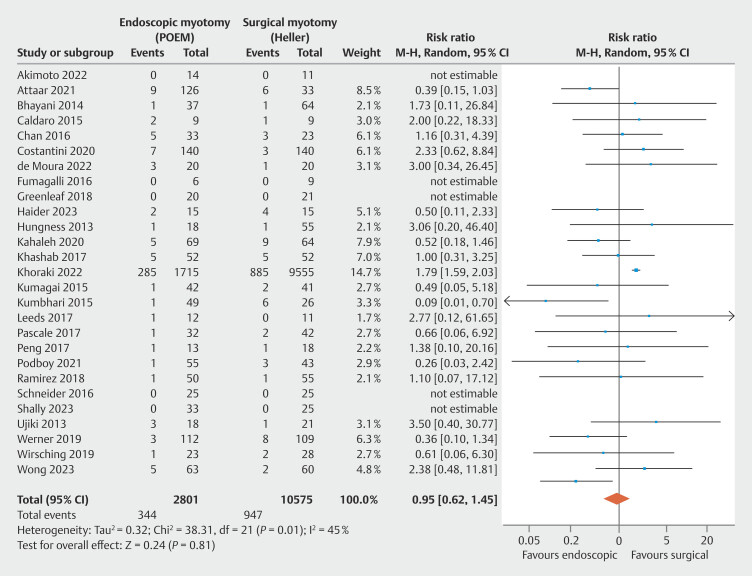
Forest plot of major adverse events (Clavien-Dindo Classification ≥ 2).

Adequate data evaluating the AEs were available from 27 studies, consisting of 25 observational studies 21 22 23 24 25 26 28 29 30 32 33 34 35 36 37 38 40 41 42 43 44 45 48 51 52 and two RCTs 9 53 , totaling 13,376 patients. No significant heterogeneity was observed among the studies. There was no difference between the groups in occurrence of major complications ( Fig. 11 : RR = 0.95; 95% CI 0.62 to 1.45; τ ^2^ = 0.32; I ^2^ = 45%; PI = 0.275 to 3.276; *P* = 0.81), supported by a low quality of evidence by GRADE ( Fig. 4 ).

## Discussion

This systematic review with meta-analysis evaluated GERD over time, comparing endoscopic versus surgical myotomy for treatment of achalasia based on updated recommendations from the Lyon Consensus 2.0 11 . Our study demonstrated that a higher incidence of GERD in early follow-up (up to 12 months) is observed in POEM when compared with HMF, with this discrepancy decreasing over time, reaching the same GERD rate between the groups in long-term follow-up (≥ 12 months). Nonetheless, there is a higher necessity for GAST in patients treated endoscopically. In evaluation of secondary outcomes, POEM demonstrated greater clinical success, shorter procedure time and hospital stay, and comparable major AEs compared with HMF.

Postoperative GERD is a primary concern involving cardiomyotomy, resulting from laxity of the cardia contingency at the esophagogastric junction. Subjective data, such as questionnaire data and nonspecific symptoms, is insufficient to make an objective and conclusive diagnosis of GERD alone, mainly because of confounding by symptoms of cardiovascular, lung, and esophageal motor diseases that resemble achalasia itself. In addition, articles included for meta-analysis that evaluated pH monitoring during follow-up followed diagnostic criteria using the DeMeester score 14 , unlike use of acid exposure time (AET) with a threshold of 6%, which has a better definition and indication for surgical treatment of GERD 54 . Consequently, the present study was based on endoscopic criteria from the Lyon Consensus 2.0 11 , a modern guideline updated in 2024. To reinforce the decision, an important confounding factor consists of partial retention of food in the esophagus of achalasia patients, giving rise to the process of acid fermentation and reduction of intraluminal pH, altering basal pHmetry. The ideal use of this test consists of manual review and comparison of results before and after cardiomyotomy, because automated assessment overestimates AET by not differentiating real reflux from fermentation 55 .

Upper endoscopy assessment was the main evidence used in our study to objectively analyze GERD according to the Lyon 2.0 consensus 11 . The 12-month time frame was the period available to divide the studies between early and late assessments and make it a viable comparison. Our results demonstrated that there is a higher rate of GERD after POEM when compared with HMF in early follow-up and confirmed the waning difference in GERD between the groups over time, reaching a statistically significant non-difference between the endoscopic and surgical approaches, results observed in previous studies 9 10 56 . This decrease in reflux over time may be attributable to the fact that achalasia generates symptoms contrary to reflux, acting as a natural barrier, and POEM initially involves acid exposure to a more sensitive esophageal mucosa, which has been affected by fermentation from previous food stasis with microscopic changes already mimicking reflux and lymphocytic esophagitis 57 . Over time, late remodeling of the esophagus resulting from more effective esophageal emptying prevents fermentation and reduces GERD in the endoscopic group 58 . Another explanation that corroborates the decrease in reflux over time could be attributed to loosening of fundoplication in the HMF group, which may progress to intrathoracic migration, recurrence of hiatal hernia, partial or complete rupture of the envelope, and failure of fundoplication 59 .

Regarding regular use of medications for GAST, indicated for symptomatic GERD, the included studies evaluated only use of proton pump inhibitors (PPI) for post-cardiomyotomy treatment. As a result, this meta-analysis evaluated the rate of regular and continuous use of PPIs, showing that patients undergoing POEM have a higher prevalence of use of this medication when compared with HMF. This fact justifies the decrease in GERD between the groups over time, because the endoscopic group has a higher prevalence of acid suppression treatment compared with the surgical group. It is worth mentioning that the ESGE recommends continuous use of acid suppression throughout life for patients undergoing POEM without fundoplication with findings of LA esophagitis grades greater than A 5 . With the advent of endoscopic full-thickness plication, which may be included in post-POEM patients, and potassium competitive acid blockers (P-CAB), which are more effective for treatment of GERD than PPIs, it is possible that the difference in GERD between POEM and HMF will decrease even further 60 61 .

An Eckardt score of ≤ 3 is widely considered to be successful treatment and, therefore, is selected to assess effectiveness of cardiomyotomy 3 9 62 . Among the studies evaluated, clinical success was greater with POEM compared with HMF. This finding aligns with arguments made by proponents of the endoscopic approach, who emphasize the advantage of preserving the integrity of the diaphragmatic hiatus as a natural anti-reflux barrier. In contrast, the surgical technique disrupts the hiatus to extend the myotomy along the esophagus, potentially increasing risk of GERD 50 51 . One reason for this finding consists of the advantage of POEM in specific achalasia phenotypes, especially achalasia type III and other esophageal spastic disorders, due to its ability to create a longer or more customized myotomy for the spastic segment through easier access to the proximal esophagus when compared with the surgical approach 50 . Greater clinical success in the endoscopic group may be correlated with the fact that most of the included studies were observational and, as a result, POEM is preferentially indicated for patients with type III achalasia whereas HMF is indicated for cases of advanced sigmoid stage achalasia, knowing that there is a higher recurrence rate in this phase 4 63 .

Procedure duration and hospital stay exhibited significant heterogeneity among the evaluated outcomes, primarily influenced by variations in physician and multidisciplinary team expertise, institutional protocols, and availability of postoperative support across the included studies. The observed disparities in procedure times may be attributed to the relative novelty of POEM compared with HMF, which inherently ties procedure efficiency to endoscopist level of experience. The present study demonstrated statistically significant reductions in procedure time and hospital stay with the endoscopic approach, supporting the notion of its less invasive nature compared with surgical methods. These findings are consistent with the recommendations outlined in the ASGE guideline 4 .

Regarding the safety profile, use of carbon dioxide (CO _2_ ) instead of ambient air significantly reduced postoperative complication rates associated with endoscopic procedures, because CO _2_ is more readily absorbed by tissues. Consequently, most reported complications, such as pneumoperitoneum and pneumomediastinum, are frequently experienced but are effectively managed intraoperatively, with minimal clinical impact during the postoperative period 64 . In this study, rates of major AEs were comparable between POEM and HMF, with both therapies considered safe and indicated as first-line treatment for achalasia 4 5 .

Despite adhering to rigorous methodological guidelines, this study has certain limitations. The primary limitation is the scarcity of high-quality data in the existing literature, including a limited number of RCTs, which increases risk of bias. Additional limitations include: absence of pH monitoring based on the updated criteria of the Lyon Consensus 2.0 11 ; limited availability of upper endoscopy in the included studies, including no subgroup analysis between patients with and without PPI use, not allowing this subgroup assessment; lack of isolated comparisons across different types of achalasia, preventing subgroup analyses; and omission of borderline GERD diagnoses, such as mild esophagitis (Los Angeles grade A) supported by histopathological scoring or electron microscopy. However, the quality limitations were mitigated by the large number of studies included, encompassing a significant patient population, which yielded statistically significant outcomes. Furthermore, assessment of post-procedure GERD was complemented by objective endoscopic criteria.

In summary, this systematic review and meta-analysis demonstrated a higher incidence of post-POEM GERD, which diminished over time, approaching similarity to HMF after 12 months, albeit with greater reliance on GAST in the endoscopic group. The higher clinical success rate, shorter procedure time and length of stay, and comparable rates of major AEs associated with POEM support the recommendations of the ASGE and ESGE guidelines endorsing both approaches as primary therapies for achalasia 4 5 . These findings support use of endoscopic treatment for achalasia in select patients, particularly those with type III achalasia, who are amenable to regular use of acid-suppressive medications.

## Conclusions

This systematic review and meta-analysis revealed a higher incidence of post-procedure GERD following POEM compared with HMF during early evaluations, with the difference diminishing over time and reaching equivalence after 12 months of follow-up, attributed to a greater reliance on regular use of gastric acid suppressors in the endoscopic group. In addition, POEM demonstrated an effectiveness and safety profile comparable to HMF.

## Supplementary Material

Supplementary Material Zusatzmaterial
